# Palpation of the Uterus Made Easier by Extension of the Rectum

**Published:** 1888-11

**Authors:** 


Palpation of the Uterus made easier by Extension of the Rectum.—According to the Vienna correspondent of the *Lancet* for June 2, 1888, a new method for facilitating the palpation of the uterus and the ovaries in cases where this mode of examination is made difficult by any peculiarity of the internal genital organs or by diseases, has been devised by Dr. Emerich Ullmann, assistant of Professor Albert. At the meeting of the Vienna Gynecological Society he suggested the introduction of a colpeurynter containing 200 to 250 cubic centimeters of water into the rectum in cases where palpation of the pelvic organs is impossible under ordinary conditions. By this application of the colpeurynter, as had been proved by Ullmann's experiments made on the cadaver and on living persons, the uterus and the ovaries are raised and brought into an anteverted position, so that, by pressing down the uterus against the touching finger, with the hand resting on the abdomen, it is possible to examine minutely from the vagina both surfaces of the uterus, the ligaments, and the ovaries, if the bladder is emptied.—*Therapeutic Gazette.*

